# Corrosion Performance in 0.5 mol/L HF Solution of Cr-Cu-Mo-Ni Porous Alloys with Varying Cr Contents

**DOI:** 10.3390/ma18174012

**Published:** 2025-08-27

**Authors:** Jiefeng Wang, Yulong Feng, Xide Li, Junsheng Yang, Wenkai Jiang

**Affiliations:** 1 School of Mechanical and Aeronautical Manufacturing Engineering, Anyang Institute of Technology, Anyang 455000, China; wangyufan2007@163.com; 2 College of Mechanical Engineering, Wuhan Polytechnic University, Wuhan 430048, China; yangjunsheng2008@163.com (J.Y.); jiangwenk@whpu.edu.cn (W.J.); 3 School of Science, Wuhan University of Technology, Wuhan 430070, China; lixide1993@163.com; 4 Hubei Key Laboratory for Engineering Structural Analysis and Safety Assessment, Wuhan 430074, China

**Keywords:** corrosion, Cr-Cu-Mo-Ni porous alloys, porosity, Cr content

## Abstract

An activation reaction sintering process was utilized to produce Cr-Cu-Mo-Ni porous alloys. Subsequently, weight loss measurements and electrochemical methods were applied to investigate the effect of Cr content ranging from 10wt% to 30wt% on the corrosive properties of Cr-Cu-Mo-Ni alloys in a 0.5 mol/L HF solution. Scanning electron microscopy (SEM) and X-ray diffraction analyses (XRD) were performed to assess the structural morphology and phase composition. As the results illustrated, Cr-Cu-Mo-Ni porous alloys possess good corrosion resistance, which is significantly higher than that of dense Ni and Cu alloys. The anti-corrosion performance of porous alloys is not proportional to the Cr content when the Cr concentration is gradually increased. When the chromium content is 20%, it exhibits the best corrosion resistance. Electrochemical measurements yielded similar results to weight loss measurements. With an increasing Cr content, double capacitive loops in electrochemical impedance spectroscopy (EIS) tests for Cr-Cu-Mo-Ni porous alloys first increased and then decreased, indicating that the corrosion process can be regulated by an electrochemical reaction. Meanwhile, after analysis, the results show that the corrosion products on the material surface adhere to the inner surface of the pores, thus improving the corrosion resistance.

## 1. Introduction

Due to their three-dimensional intersecting pore structures, porous materials are widely used in fields such as separation and filtration, sound absorption and noise reduction, energy, biomedical engineering, pre-dedusting, antifouling, and anti-corrosion [1,2,3,4]. Porous alloys, owing to their excellent fluid/gas permeability, high mechanical strength, and advantages in mechanical processing and welding performance, play an indispensable role in various industries, especially under harsh service conditions [5,6,7,8]. In the field of anti-biofouling and corrosion protection, aiming at the problem of insufficient long-term stability of SLIPSs (slippery liquid-infused porous surfaces), Meng et al. [4] constructed a micro-nano bimodal porous structure and systematically studied the effects of different spraying powers on coating performance. After 20 days of dynamic immersion, SLIPS-27 exhibited a lubricant retention rate of 72.6%, with the sliding angle only increasing from 5° to 16°. The corrosion current density of SLIPS-27 (0.26 μA/cm^2^) was two orders of magnitude lower than that of the bare substrate, while the charge-transfer resistance (Rct) was two orders of magnitude higher, maintaining excellent protective capability after 10 days of immersion, thus enhancing its long-term stability and durability. It also provides a reference for long-term anti-biofouling and corrosion protection efforts in marine engineering. Powder metallurgy technology allows for the preparation of traditional porous alloys using either elemental powders or pre-alloyed powders. However, processing with pre-alloyed powders as starting materials requires complex and expensive procedures. In contrast, elemental powders are ideal for large-scale manufacturing and industrial applications due to their low cost and abundant availability as raw materials [8,9].

Various highly corrosive acids and inorganic salts, such as concentrated phosphoric acid, sulfuric acid, dilute hydrofluoric acid, and calcium sulphate precipitation, are involved in the wet phosphoric acid process [10]. Although hydrofluoric acid is a weak acid with low concentration, it is no less destructive in the aggressive penetration of its fluoride ions than many other strong acids [9]. As a result, conventional porous alloys cannot survive the hostile environment and lose their functional properties under severe conditions. Therefore, there is an urgent need to develop novel porous alloys for the wet-process phosphoric acid industry.

The porous Cr-Cu-Mo-Ni alloy was successfully fabricated by powder metallurgy techniques to explore filter materials applicable in the wet-process phosphoric acid industry. The study of this porous alloy is inspired by its compressed counterpart, which exhibits excellent corrosion resistance in multiple acids, including hydrofluoric acid [11,12,13]. Previous research [14,15,16,17] investigated the corrosive characteristics of Cr-Cu-Mo-Ni porous alloys in other acidic environments. In addition, studies have demonstrated that the Cr element enhances metal corrosion resistance [18].

Therefore, the corrosion behaviour of Cr-Cu-Mo-Ni porous alloys with Cr content 10–30wt% in a 0.5 mol/L HF solution was studied. Furthermore, the composition and microstructure of the porous Cr-Cu-Mo-Ni alloy were analyzed by X-ray diffraction (XRD) and scanning electron microscopy (SEM), and the corrosion products were characterized by X-ray photoelectron spectroscopy (XPS). Moreover, electrochemical impedance spectroscopy (EIS) and polarization curves were used to evaluate the corrosion processes occurring in Cr-Cu-Mo-Ni porous alloys and study their corrosion mechanism.

## 2. Materials and Methods

### 2.1. Preparation and Characterization of Cr-Cu-Mo-Ni Porous Alloys

An activation reaction sintering technique was introduced to synthesize Cr-Cu-Mo-Ni porous alloys using Cr, Cu, Mo, and Ni elemental powders as raw materials (Nangong County Metal Powder Co., Ltd., Hebei Province). All of the selected powders utilized in this study were 38~74 μm in diameter; (0.1 + x) Cr0.05Cu0.1Mo (0.75 − x)Ni (x is equal to 0, 0.05, 0.1, 0.15, 0.2) were the content proportions of Cr-Cu-Mo-Ni porous alloys. Additionally, the powders were mixed in a planetary ball mill (Manufacturer: Nanjing Nanda Instrument Co., Ltd. Instrument Model: QM-QX2) for 5 h at a ball-to-powder ratio (BPR) of 1:5 and a rotational speed of 200 rpm under an air atmosphere. The mixed powders were compacted under a pressure of 160 MPa into a green compact with a diameter of 25 × 10 × 2 mm. Subsequently, the specimens were sintered in a vacuum furnace (Manufacturer: Hunan Xubo Metallurgical Equipment, Instrument Model: XBZK-150) at 1150 °C for 2 h and then cooled to room temperature in the furnace [15]. Finally, it was cut into 1 mm × 1 mm square blocks by wire cutting, then ultrasonically cleaned with absolute ethanol (China National Pharmaceutical Group Corporation) multiple times, dried at 60 °C in a vacuum drying oven, and finally became the sample for XRD testing. The test parameters were as follows: tube voltage of 40 kV, tube current of 40 mA, continuous scan speed of 5°/min, and angle range of 5°–80° (2θ). The model of the testing instrument is Siemens D500 X-ray (Siemens Healthineers, Forchheim, Germany).

X-ray diffraction was then utilized to ascertain the structure of the manufactured Cr-Cu-Mo-Ni porous alloy electrode (XRD). The Archimedes technique was applied for assessing different samples’ porosity as depicted in the following formula:

(1) $$P=\frac{V_{1}-(M_{1}/\rho_{1}+M_{2}/\rho_{2}+M_{3}/\rho_{3}+M_{4}/\rho_{4})}{V_{1}}\times 100\%$$

where P denotes the porosity; V_1_ is the overall sample size; M_1_, M_2_, M_3_, M_4_, ρ_1_, ρ_2_, ρ_3,_ and ρ_4_ correspond to the mass and densities of Ni, Cr, Mo, and Cu elements.

### 2.2. Immersion Test

The variation in the corrosion rate of Cr-Cu-Mo-Ni porous alloys with Cr contents ranging from 10wt% to 30wt% at room temperature (20 °C) was investigated. The Cr-Cu-Mo-Ni porous alloy samples were placed statically in a 0.5 mol/L hydrofluoric acid solution (China National Pharmaceutical Group Corporation) without any agitation during the process. A curve was plotted by measuring the relative mass loss as a function of corrosion time in the 0.5 mol/L hydrofluoric acid solution. Subsequently, the specimens were ultrasonically rinsed in distilled water for 30 min. Subsequently, they were dried in a vacuum drying oven at 60 °C for 60 min and then weighed on an analytical balance with a resolution of 0.0001 g. Finally, SEM was utilized to analyze the morphology of Cr-Cu-Mo-Ni porous alloy electrodes prior to and following immersion.

### 2.3. Electrochemical Measurement

#### 2.3.1. Preparation of Corrosion Test Samples

A copper wire was linked to one side of the Cr-Cu-Mo-Ni porous alloys at mid-height to make an electrical connection. The electrical connection was tested using a multimeter. The specimens were then enclosed in an insulating rubber that had to dry for 24 h at rtp prior to usage. Prior to pouring the insulating rubber on the samples, a polytetrafluoroethylene tape was utilized to conceal the non-test zone which may prevent the electrode from leaking due to long-term testing. The exposure area was 1 cm^2^.

#### 2.3.2. Electrochemical Test Procedure

A classic three-electrode system was used for the electrochemical tests. The Cr-Cu-Mo-Ni porous alloy served as the working electrode, with both sides exposed to the electrolyte. The reference electrode was an Ag/AgCl electrode in a 3 M KCl solution, and the counter electrode was a platinum sheet with the same surface area. The potentiodynamic polarization curves were measured within a potential range of −0.8 V to 1.0 V (vs. Ag/AgCl) at a scanning rate of 1 mV/s. The test electrolyte was a 0.5 mol/L hydrofluoric acid solution (analytical reagent).

EIS as well as potentiodynamic polarization tests were conducted after 10 h immersion in an open circuit. Three samples with different contents were tested, and each sample was tested in triplicate. EIS measurements were carried out by open circuit potential (OCP) at 7 points per decade with a frequency range from 10^5^ Hz to 10^−2^ Hz. After that, the programme Zview2.0 was used to evaluate the EIS spectra.

## 3. Results and Discussion

### 3.1. Characterization of Porous Cr-Cu-Mo-Ni Alloy

**Figure 1:**
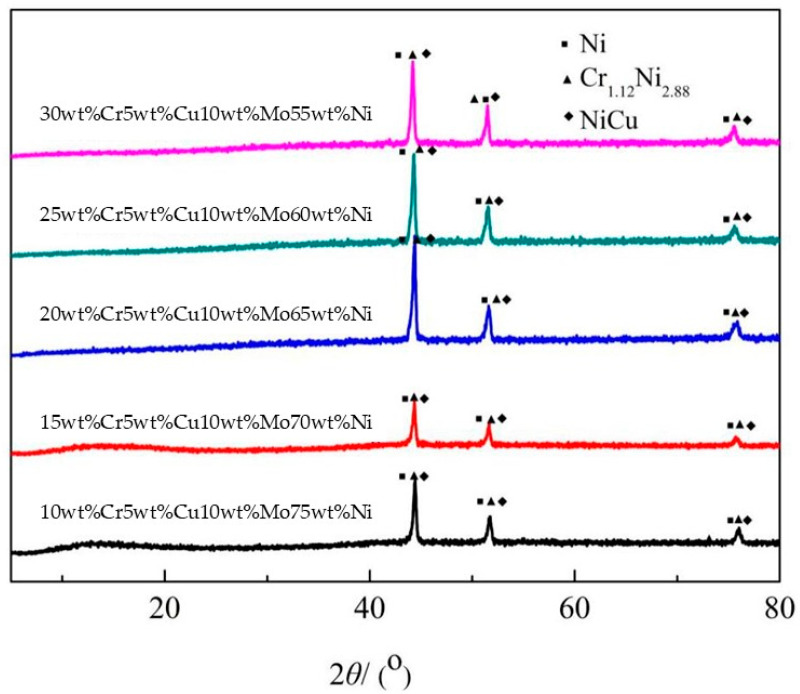
XRD pattern of porous CrCuMoNi alloys prepared with different Cr contents.

The XRD measurements of Cr-Cu-Mo-Ni porous alloys with varying Cr concentrations were performed to confirm the phase compositions, as shown in Figure 1. Mo has no significant diffraction peak for Cr-Cu-Mo-Ni porous alloys, as indicated by XRD patterns, suggesting an amorphous structure [19,20]. In the XRD spectrum, the three characteristic intense peaks assigned at diffraction angles with 2θ at 44.4°, 51.6°, and 76° are Ni, Cr_1.12_Ni_2.88_, and NiCu, respectively. Peak intensities improve as Cr content increases compared to 10wt%Cr concentrations, which means that more Cr interacts with Ni to form Cr_1.12_Ni_2.88_, and the alloying level of the final sintered species increases with increasing Cr content.

**Figure 2:**
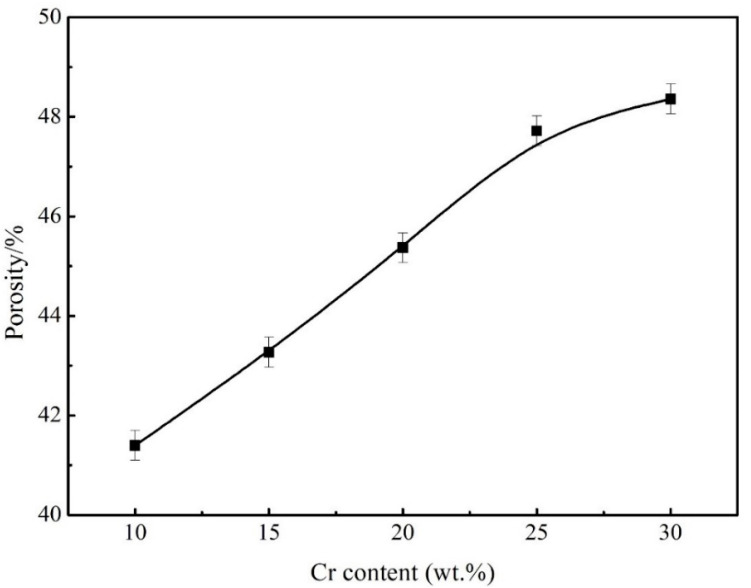
Porosity of Cr-Cu-Mo-Ni porous alloys with different Cr contents.

The permeability of Cr-Cu-Mo-Ni porous alloys is shown in Figure 2. The porosity varies from 41.4 percent to 48.36 percent as the Cr content increases. As is well known, during the pressing process, microscopic holes emerge in the green compact, and these pores are relatively small. Furthermore, owing to differences in diffusion rates across elements, the Kirkendall effect promotes Cr, Mo, and Cu atoms to move in the direction of Ni atoms. Pores formed at the original sites of the Cr, Mo, and Cu atoms. The diffusion rate of Cr atoms in mixed elements increased as the temperature approached 1150 °C and the Cr concentration increased, favouring the formation of Kirkendall pores [21,22].

### 3.2. Immersion Test

**Figure 3:**
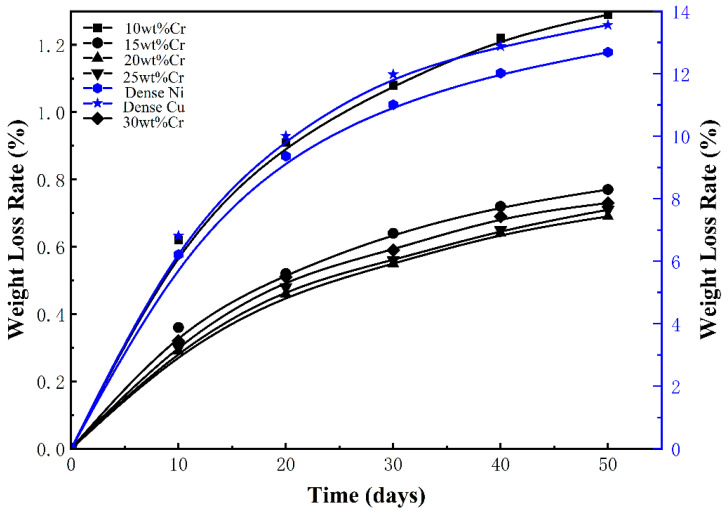
The effect of Cr content on the weight loss rate of porous CrCuMoNi alloys in a 0.5 mol/L HF solution (20 °C).

The mass variations after immersing in a 0.5 mol/L HF solution for 50 days are shown in Figure 3. After 50 days of corrosion, the weight changes in dense Ni and Cu are 12.69% and 13.56%, respectively, while the weight loss rate of porous alloys with Cr content ranging from 10% to 20% is 1.29% and 0.69%, respectively. And the weight loss rate rises to 0.73% when the Cr concentration reaches 30%, which is significantly less than dense Ni and Cu. In addition, the changes in pore structure parameters show a similar trend (as shown in Table 1), first decreasing and then increasing. Among them, the 20wt%Cr5wt%Cu10wt%Mo65wt%Ni porous alloy has an average pore size of 0.68 μm and a porosity of 5.2%. When the average pore size is small and the permeability is low, it is difficult for the corrosive media to penetrate into the interior of the material, which reduces the contact area with the internal matrix and may slow down the corrosion rate. Considering factors such as Cr content, oxides are easily formed on the surface after corrosion. Through the combined action of chromium and oxygen, even if the average pore size is small, the penetration of corrosive media can be further hindered, thereby enhancing the corrosion resistance.

**Figure 4:**
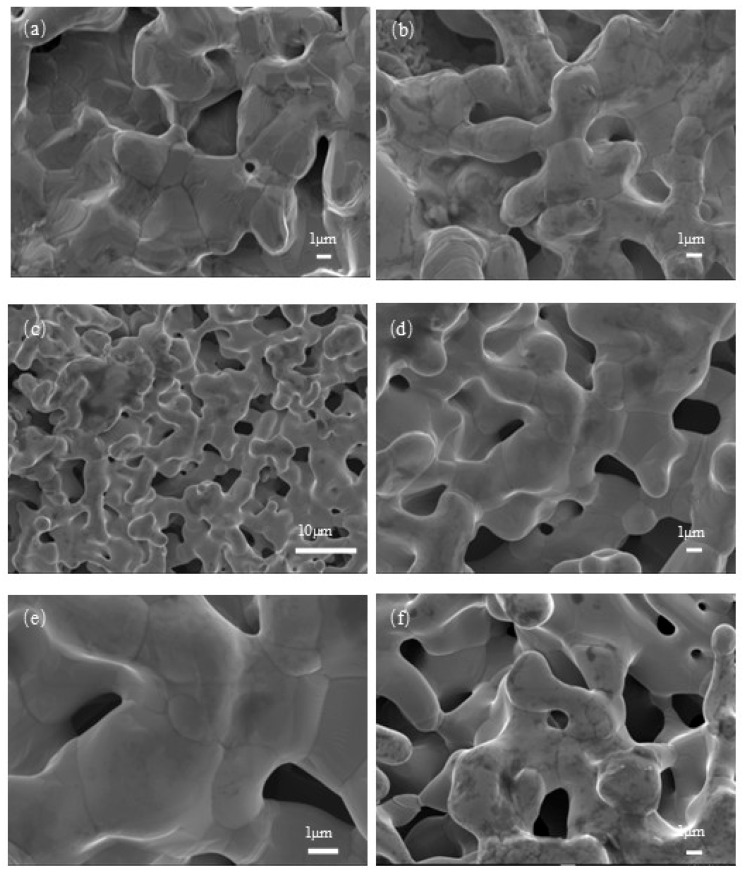
The scanning electron microscopy (SEM) images of Ni-Cr-Mo-Cu alloys with different Cr contents before and after corrosion in a hydrofluoric acid (HF) solution at 20 °C for 50 days. (**a**,**b**) Images of 10wt%Cr before and after corrosion. (**c**,**d**) Images of 20wt%Cr before and after corrosion. (**e**,**f**) Images of 30wt%Cr before and after corrosion.

Figure 4 presents the scanning electron microscopy (SEM) images of Cr-Cu-Mo-Ni porous alloys with different Cr contents before and after corrosion. Figure 4c,d show the SEM images of the Cr-Cu-Mo-Ni porous alloy with a Cr content of 20wt% before and after immersion. In Figure 4c, before immersion, the surface of this porous alloy is smooth, and the porous channels are closely connected, indicating that the alloying process of Cr-Cu-Mo-Ni is complete. The pore structure of the green compact is composed of interstitial pores formed between powder particles, and powder metallurgy technology provides an ideal method for controlling the structure of porous alloys. Correspondingly, compared with the material before the immersion test, the pore structure of the porous alloy with a Cr content of 20wt% does not change significantly, and there are almost no corrosion pits on the surface of the sample (Figure 4d). Figure 4a,b show the SEM images of the Cr-Cu-Mo-Ni porous alloy with a Cr content of 10wt% before and after immersion, and Figure 4e,f show the SEM images of the Cr-Cu-Mo-Ni porous alloy with a Cr content of 30wt% before and after immersion. Comparing the Cr-Cu-Mo-Ni porous alloys with Cr contents of 10wt% and 30wt%, more black spots appear after immersion. These may be tiny blind holes or oxide layers with extremely low Cr content, resulting in insufficient local oxidation and a loose structure. Therefore, the porous alloy with a Cr content of 20wt% exhibits good corrosion resistance in a 0.5 mol/L hydrofluoric acid (HF) solution.

### 3.3. X-Ray Photoelectron Spectroscopy

**Figure 5:**
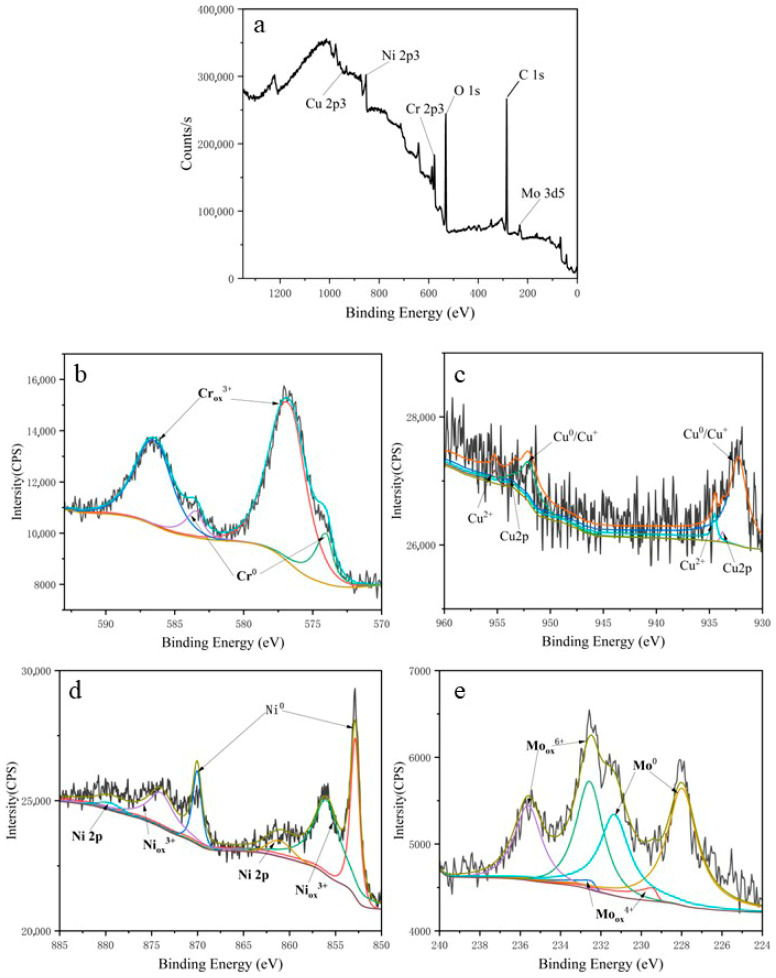
XPS spectra of Cr-Cu-Mo-Ni porous alloys after corrosion tests: wide scan, Ni-2p, Cr-2p, Mo-3d, and Cu-2p ((**a**–**e**), respectively).

X-ray photoelectron spectroscopy (XPS) was employed to characterize the chemical states of elements in the Cr-Cu-Mo-Ni porous alloy. As shown in Figure 5, this figure presents the XPS spectrum of the surface of the Cr-Cu-Mo-Ni porous alloy electrode, measured after the electrode was immersed in a 0.5 mol/L hydrofluoric acid (HF) solution. Among them, Figure 5a is the wide-scan spectrum of this surface, indicating the presence of six elements: Ni, Cr, Mo, Cu, O, and C. Meanwhile, Figure 5b shows the deconvolution results of Cr 2p, corresponding to metallic Cr (binding energy: 574 eV), oxidized state (binding energy: 576.7 eV), and Cr(OH)_3_ (binding energy: 577 eV), respectively. After the corrosion of the Cr-Cu-Mo-Ni porous alloy, corrosion products adhere to the inner surface of the pores and are strengthened through the synergistic effect of chromium and oxygen, thereby improving the corrosion resistance of the porous alloy; moreover, the XPS analysis results are consistent with those of the immersion test. Figure 5c shows the deconvolution results of Cu 2p, corresponding to metallic Cu (binding energy: 932.27 eV) and oxidized CuO (binding energy: 934.5 eV), respectively. Since the binding energy of Cu^+^ is extremely close to that of metallic Cu, its existence state cannot be clearly identified. In acidic media such as hydrofluoric acid (HF), Cu exhibits high thermodynamic stability and a slow dissolution rate. Its corrosion products (e.g., CuO) may form a loose but continuous coating on the pore surface, which, to a certain extent, hinders the direct erosion of the alloy matrix by HF solution and helps reduce the overall corrosion rate. Figure 5d is the narrow-scan spectrum of Ni 2p, corresponding to metallic Ni (binding energy: 852.8 eV), oxidized NiO (binding energy: 855.94 eV), and a satellite peak (binding energy: 861.08 eV), respectively. This result is consistent with the previous research conclusion that “the surface of Ni-based alloys is dominated by NiO” [23]. Figure 5e is the narrow-scan spectrum of Mo 3d, which can be fitted into four characteristic peaks, corresponding to metallic Mo (binding energy: 227.97 eV), oxidized MoO_3_ (binding energy: 232.56 eV), and MoO_2_ (binding energy: 229.43 eV), respectively. This indicates that most of the molybdenum in the alloy exists in the form of oxides. This prevents the HF solution from penetrating into the matrix through pores and significantly reduces the corrosion rate. A Mo content of 10% can ensure the coverage density of the oxidation products.

X-ray photoelectron spectroscopy (XPS) analysis indicated that after the immersion corrosion test, Ni, Cr, Cu, and Mo in the Cr-Cu-Mo-Ni porous alloy electrode all existed in the form of metal compounds. This is mainly attributed to the fact that the surface product of the sample after corrosion is chromium oxide, and this process may be initiated by the following reaction [24]:

(2) $$2Cr+3H_{2}O\to Cr_{2}O_{3}+6H^{+}+6e^{-}$$

Since the main corrosion products of the sample are oxides, the presence of these corrosion products retards or slows down the corrosion process under the combined action of chromium and oxygen. In addition, the formation of Mo-based oxides on this surface can also improve corrosion resistance, with the relevant chemical reactions as follows [14]:

(3) $$Mo+2H_{2}O\to MoO_{2}+4H^{+}+4e^{-}$$

The further oxidation of Mo-based oxides to molybdenum trioxide (MoO_3_), combined with the synergistic effect of chromium oxides, further enhances the pitting resistance of the alloy system [25]. Owing to the presence of Cr and Mo components, Cr-Cu-Mo-Ni porous alloys exhibit superior corrosion resistance compared to pure Ni, and this resistance improves as the concentration of these elements increases. On the other hand, as the Cr content increases, Table 1 shows that the average pore size and permeability of the samples first decrease and then increase. When the Cr content is relatively low, it may be more difficult to resist the continuous corrosion of the rapidly penetrating medium due to the inability to form a sufficient amount of oxides. As the Cr content increases, the amount of post-corrosion products increases, which leads to a decrease in average pore size and permeability, thereby improving the corrosion resistance. When the Cr content reaches 30wt%, the porosity also increases accordingly; this causes an increase in specific surface area, which in turn accelerates the corrosion rate [26]. Considering these two aspects comprehensively, the Cr-Cu-Mo-Ni porous alloy containing 20wt%Cr exhibits superior corrosion resistance in a 0.5 mol/L hydrofluoric acid (HF) solution.

### 3.4. Electrochemical Measurements

#### 3.4.1. Open Circuit Potential

**Figure 6:**
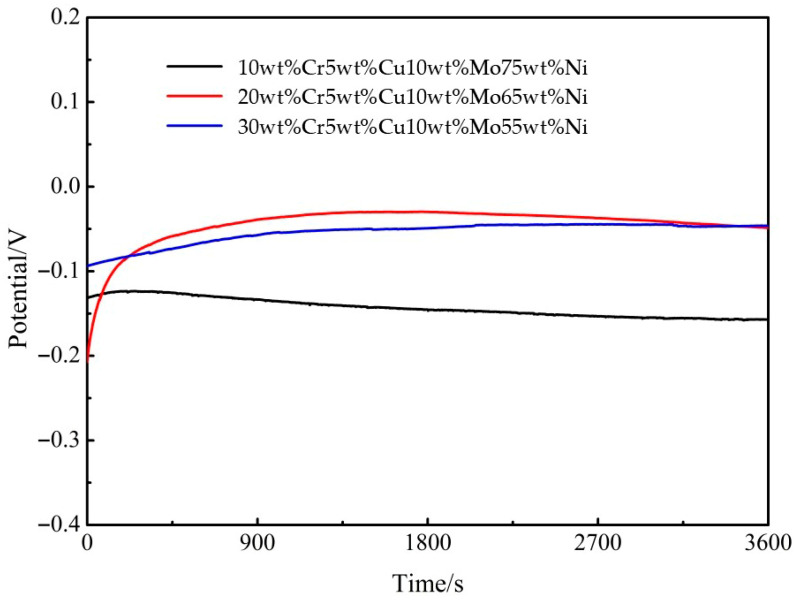
Relationship between porous material composition and open circuit potential.

Figure 6 depicts the open circuit potential as a function of time. As demonstrated, the open circuit potential of Cr-Cu-Mo-Ni porous alloys varies expeditiously and then stabilizes after a brief period. A shift in open circuit potential is indicative of the chemical stability of the tested electrode. Furthermore, it is generally established that anti-corrosion materials have a higher positive charge and greater corrosion resistance. Increasing positive open circuit potential of Cr-Cu-Mo-Ni porous alloys with the increased Cr contents was reported in this study [22]. Because of this phenomenon, samples with a greater Cr content exhibit enhanced corrosion resistance.

#### 3.4.2. Electrochemical Impedance Spectroscopy (EIS)

**Figure 7:**
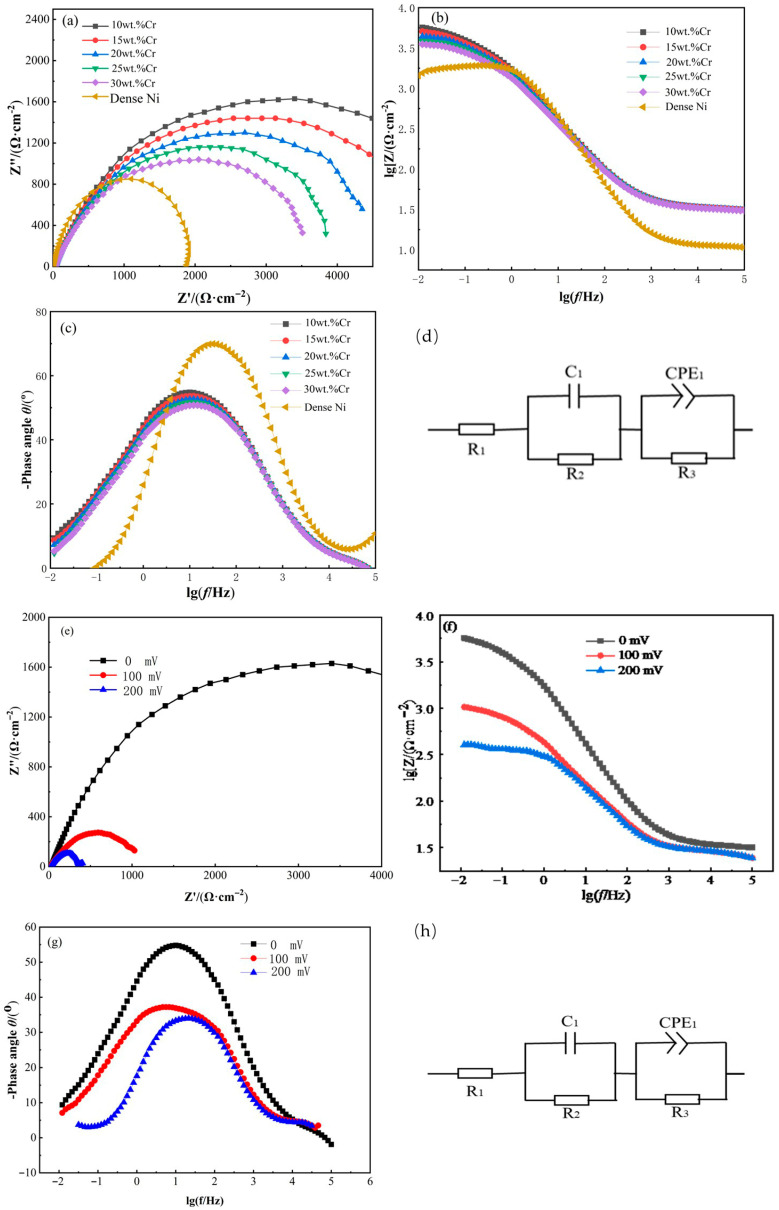
Nyquist plots and Bode plots of Cr-Cu-Mo-Ni porous alloys at different Cr contents (**a**–**d**) and different open circuit potentials of Cr-Cu-Mo-Ni porous alloy with 20wt%Cr (**e**–**h**): (**a**,**e**) Nyquist plots; (**b**,**f**) lg Z vs. lg f; (**c**,**g**) phase angle vs. lg f; (**d**,**h**) equivalent circuit.

Corrosion resistance as well as the nature of oxide layer development in corrosive environments are both evaluated using EIS, which is a frequently used method. When Cr-Cu-Mo-Ni porous alloy electrodes were immersed in a 0.5 mol/L HF solution for 10 h, the impedance spectrum of the electrodes was measured. The results are presented in Figure 7, in which all the specimens demonstrate similar spectral characteristics.

As observed in Nyquist plots (Figure 7a), variations in Cr content have a significant impact on the diameter of the semi-circle, and the diameters of semi-circular curves of 20wt%Cr Cr-Cu-Mo-Ni porous alloy are larger than those of other samples. Nyquist graphs have semi-circular curves centred under the x-axis, which aids in the comprehension of charge transfer on uneven surfaces, and increasing Cr improves corrosion behaviour. In the medium-to-low frequency range (Figure 7b,c), capacitive behaviour is observed, characterized by a phase angle approaching the maximum angle typical of passive materials. This indicates that a substance is generated on the sample surface in the electrolyte, which acts synergistically with oxygen. Additionally, a prominent phase-angle peak might suggest the interaction of two time constants. The corrosion resistance of the Cr-Cu-Mo-Ni porous alloy electrode is examined using an equivalent circuit. The objective of this corresponding circuit is to replicate the EIS data obtained from the experiment, as depicted in Figure 7d. As shown in the figure, in the electrical equivalent circuit, a pair of capacitive and resistive components are connected in series with the solution resistance (R_s_). Here, R_2_ represents the additional resistance of the solution within the pores, R_3_ denotes the charge-transfer resistance of the oxygen layer, C_1_ indicates the capacitance of the pore walls, and CPE_1_ represents the capacitance of the oxygen layer [27].

It should be emphasized that the circuit model employed in this study does not precisely mirror the actual system. To account for the heterogeneity of the samples, a constant-phase element (CPE) was utilized instead of a traditional capacitor [28,29]. Table 2 presents the EIS results after fitting the data to the circuit model. The R values of the Ni20wt%Cr10wt%Mo5wt%Cu porous alloy are higher compared to other samples, and these variations are consistent with the results of the immersion test. These findings suggest that alloys containing 20wt%Cr exhibit higher corrosion resistance.

In contrast to the Nyquist, Bode, and equivalent circuits shown in Figure 7e–h, Table 3 shows the results obtained from the EIS test after fitting with the relevant circuit. The R_3_ values decline as the voltage increases, indicating that the specimens exhibit good corrosion resistance at the open circuit potential. Moreover, the R_s_ values are negligible at different voltages, demonstrating that the diffusion process was merely a secondary phase [26].

#### 3.4.3. Polarization Test

**Figure 8:**
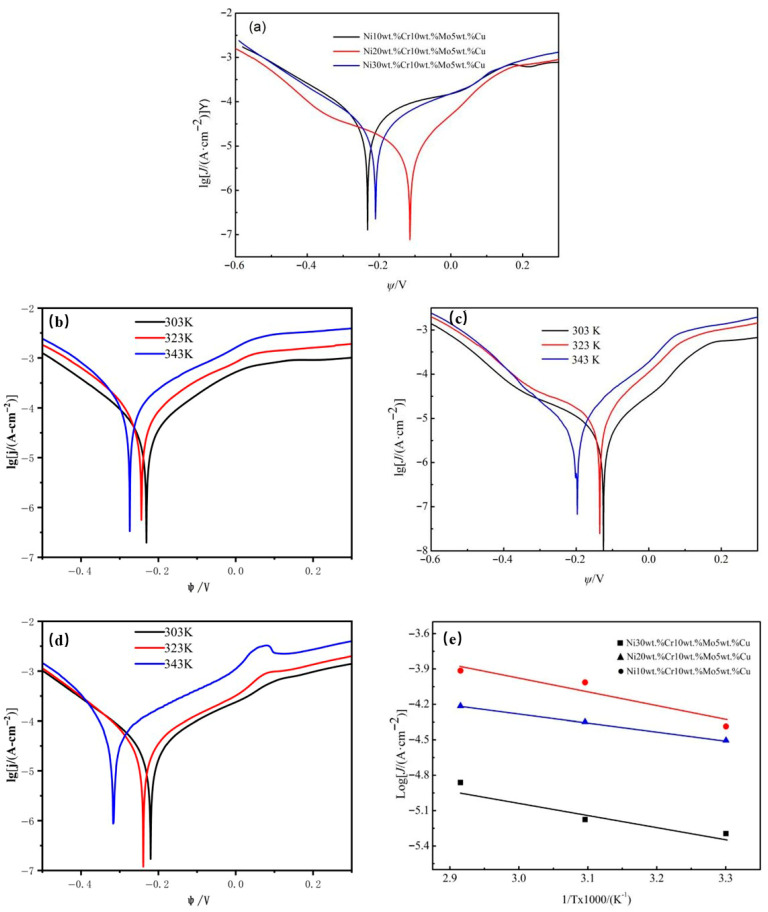
Polarization curves of Cr-Cu-Mo-Ni porous alloys: (**a**) Polarization curves at different Cr contents. (**b**) Polarization curves at different temperatures of 10wt%Cr porous alloy. (**c**) Polarization curves at different temperatures of 20wt%Cr porous alloy. (**d**) Polarization curves at different temperatures of 30wt%Cr porous alloy. (**e**) Arrhenius curves.

At room temperature (293 K), Figure 8a illustrates the important changes in potentiodynamic polarization for Cr-Cu-Mo-Ni porous alloys with varying Cr contents, and Table 4 lists the calculated electrochemical parameters. The corrosion current density (J_corr_) is established by extrapolating the linear Tafel portion of the anodic and cathodic polarization curves [30], and it appears to be more precise than free corrosion potential (φ_corr_) values [22]. Additionally, a material with low corrosion potential and relatively obvious corrosion current density, and a higher polarization resistance (R_p_) would oxidize and corrode more readily. The 20wt%Cr porous alloy electrode possesses a relatively higher free corrosion potential, a lower polarization resistance, and a lower corrosion current density. Therefore, this alloy electrode demonstrates excellent corrosion resistance in a 0.5 mol/L HF solution. The results are in accordance with the immersion test.

Figure 8b–d show the polarization curves of porous alloy electrodes with Cr contents of 10%, 20%, and 30% at different temperatures. As illustrated, the porous alloy electrode with 20wt%Cr exhibits the strongest corrosion resistance. With increasing temperature, the corrosion potential (φcorr) values shift to more negative potentials, and the slopes of the cathodic region become nearly parallel. Table 5 summarizes the data obtained from the 20wt%Cr porous alloy, showing that the corrosion current density (Jcorr) increases with rising temperature. This indicates that the reduction of oxygen on the metal surface follows the same activation mechanism, and the defect rate of the corrosion product layer increases with temperature.

The activation energy of the corrosion process can be calculated by applying the Arrhenius relationship and the equation presented below [31]:

(4) $$Logj_{0}=Log\;A-E_{a}/(2.303R\cdot T)$$

E_a_, R, A, as well as T were the “apparent activation corrosion energy, the gas constant, the Arrhenius preexponential constant, as well as the absolute temperature”. The E_a_ values for the samples with varying Cr concentrations were determined using the Arrhenius plots illustrated in Figure 8e. The E_a_ values for the samples with varying Cr concentrations are reported in Table 4. The porous alloy electrode with 20wt%Cr has a higher activation energy during the corrosion process. This indicates that the product formed on the electrode surface can prevent corrosive species from accessing the electrode, thereby reducing its corrosion rate in a 0.5 mol/L hydrofluoric acid (HF) solution.

## 4. Conclusions

The corrosion performance of Cr-Cu-Mo-Ni porous alloys in a 0.5 mol/L hydrofluoric acid solution was investigated by means of a weight loss method and electrochemical analysis. Previous studies support the following inferences:

(1) The corrosion products obtained after the corrosion of Cr-Cu-Mo-Ni porous alloys in a 0.5 mol/L hydrofluoric acid solution adhere to the inner surface of the pores, and the corrosion resistance is improved through the combined action of chromium and other elements.
(2) Cr-Cu-Mo-Ni porous alloys with different Cr contents all exhibit good corrosion resistance in a 0.5 mol/L hydrofluoric acid solution.
(3) Compared with dense Ni alloys and Cu alloys, the Cr-Cu-Mo-Ni alloys with a porous structure have a slower corrosion rate. With the increase in Cr content, the weight loss rate of the porous alloy first decreases and then increases. Among them, the corrosion resistance of the Cr-Cu-Mo-Ni porous alloy containing 20wt%Cr is significantly improved compared with those containing 10wt%Cr and 30wt%Cr.

## Figures and Tables

**Table 1:** The change rate in the pore structure of Cr-Cu-Mo-Ni porous alloys after the immersion test.

Samples	Average Aperture (μm)	Permeability (%)
10wt%Cr5wt%Cu10wt%Mo75wt%Ni	3.12	7.2
15wt%Cr5wt%Cu10wt%Mo70wt%Ni	2.03	6.6
20wt%Cr5wt%Cu10wt%Mo65wt%Ni	0.68	5.2
25wt%Cr5wt%Cu10wt%Mo60wt%Ni	0.85	5.8
30wt%Cr5wt%Cu10wt%Mo55wt%Ni	1.63	6.4

**Table 2:** Fitting results of EIS measurements at various Cr contents on the investigated Cr-Cu-Mo-Ni porous alloy.

Porous Cr-Cu-Mo-Ni	Cr Content (wt%)				
	10	15	20	25	30
R_1_ (Ω)	30.63	30.95	31.99	31.59	31.73
C_1_ (mF)	2.52 × 10^−5^	2.70 × 10^−5^	1.58 × 10^−5^	1.64 × 10^−5^	0.000219
R_2_ (Ω)	2.90 × 10^−5^	1.86 × 10^−5^	1.30 × 10^−5^	5.53 × 10^−5^	0.132
CPE1-T	0.000178	0.000169	0.000145	0.000154	0.000150
CPE1-P	0.66502	0.66959	0.68314	0.67698	0.68571
R_3_ (Ω)	3684	4068	5757	5172	4093

**Table 3:** Fitting results of EIS measurements at various external voltages vs. open circuit potential on the investigated Cr-Cu-Mo-Ni porous alloy with 20wt%Cr.

Porous Cr-Cu-Mo-Ni Alloys	External Voltages (mV)		
	0	100	200
*R*_1_ (Ω)	31.99	21.03	26.71
*C*_1_ (mF)	1.58 × 10^−5^	2.37 × 10^−7^	5.70 × 10^−5^
*R*_2_ (Ω)	1.30 × 10^−5^	5.272	0.4834
*CPE*_1_ *-T*	0.000145	0.00065016	0.00046128
*CPE*_1_ *-P*	0.68314	0.5705	0.63847
*R*_3_ (Ω)	5757	1108	258.8

**Table 4:** Corrosion parameters obtained from potentiodynamic polarization curves (303 K).

Porous Cr-Cu-Mo-Ni Alloys	*φ*_corr_/V	*J*_corr_/(A·cm^−2^)	*R_p_*/Ω	*E_a_*/(kJ/mol)
10wt%Cr5wt%Cu10wt%Mo75wt%Ni	−0.232	4.113 × 10^−5^	833	14.55
20wt%Cr5wt%Cu10wt%Mo65wt%Ni	−0.112	5.066 × 10^−5^	8241	23.74
30wt%Cr5wt%Cu10wt%Mo55wt%Ni	−0.21	3.131 × 10^−5^	1198	21.43

**Table 5:** Corrosion parameters obtained from potentiodynamic polarization curves of 20wt%Cr porous alloy at different temperatures.

Temperature/K	*φ*_corr_/V	*J*_corr_/(A·cm^−2^)	*R_p_*/Ω	Passivation Range/V
303	−0.123	6.339 × 10^−6^	6021	0.10
323	−0.137	6.673 × 10^−6^	4430	0.06
343	−0.198	1.376 × 10^−5^	2718	0.04

## Data Availability

The original contributions presented in this study are included in the article. Further inquiries can be directed to the corresponding author.
